# Successful management of primary non Hodgkins lymphoma of the cranial vault

**DOI:** 10.4314/pamj.v8i1.71168

**Published:** 2011-04-30

**Authors:** Zouhour Fadoukhair, Issam Lalya, Mounia Amzerin, Basma Elkhanoussi, Yassir Sbitti, Saber Boutayeb, Hind M’rabti, Noureddine Benjaafar, Hassan Errihani

**Affiliations:** 1 Department of Medical Oncology, National Institute of Oncology, Rabat, Morocco; 2 Department of Radiation oncology, National Institute of Oncology, Rabat, Morocco; 3 Department of pathology, National Institute of Oncology, Rabat, Morocco; 4 Department of Medical Oncology, Military Instruction Hospital Mohammed V, Rabat, Morocco

**Keywords:** B-Cell lymphoma, primary bone lymphoma, extranodal lymphoma, cranial vault

## Abstract

Primary bone lymphoma (PBL) is a relatively uncommon entity. However, involvement of the cranial vault is an unusual manifestation of aggressive non-Hodgkin’s lymphoma. We report the case of a 42-year old immunocompetent woman who presented with an enlarging mass involving the right parietal bone. Magnetic resonance imaging (MRI) of the brain revealed an expansive tumor that affects the right parietal bone. Computed tomographic (CT) scans of the abdomen, chest and pelvis were negative for lymphadenopathy or organomegaly. Biopsy of the mass showed diffuse large B-cell non-Hodgkin’s lymphoma confirmed by immunohistochemical study. The patient had a complete response after 4 cycles of chemotherapy followed by external beam radiotherapy. After a follow-up of more than 9 months the patient is still in good local control without distant metastasis. The aim of our work is to report a case of Primary bone lymphoma of the cranial vault with good response to treatment combining sequential chemotherapy and radiotherapy.

## Background

Primary bone lymphoma (PBL) represents about 5% of all non-Hodgkin lymphomas (NHL) and 3% of all bone malignancies [1]. The femur, tibia and pelvis are the most common skeletal sites [1]. To our knowledge, only fourteen immunocompetent patients with primary cranial vault lymphoma have been reported in the literature [2-15]. Because of the rarity of this entity and the lack of evident literature, its optimal management is still unknown. We report a case of good response to treatment combining sequential chemotherapy and radiotherapy. We report a case of primary bone lymphoma of the cranial vault with good response to treatment combining sequential chemotherapy and radiotherapy.

## Case presentation

A 42-year old immunocompetent woman presented with a 10-month history of enlarging mass involving the right parietal bone without history of trauma. Physical examination showed a localized mass of 8 × 6 cm in diameter, in the right parietal region. There was no erythema and it was firm and slightly tender. The remainder of the examination was unremarkable; in particular no lymphadenopathy and no abdominal masses were felt.

**Figure 1:**
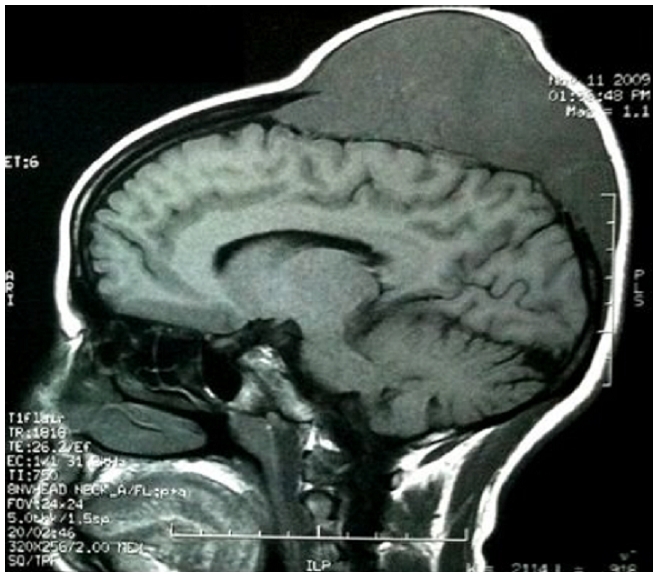
Sagittal T1 flair showing an expansive tumor that affects the right parietal bone in a 42-years old patient with primary non Hodgkin's lymphoma of the cranial vault

Neurological examination was normal. MRI of the brain revealed an expansive tumor (11 × 5 cm) affecting the right parietal bone, including the scalp (Figure 1). There was no evidence of parenchymal involvement. CT scans of the abdomen; chest and pelvis were negative for lymphadenopathy or organomegaly. There was no evidence of bone marrow involvement.

**Figure 2:**
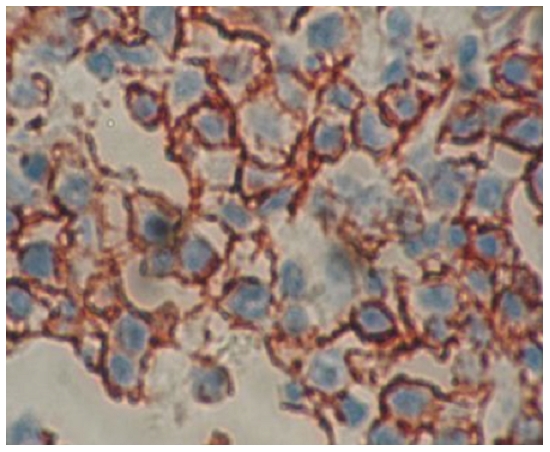
Diffuse large B cell lumphoma positive for CD20 (immunohistochemistry CD20 orginal magification ×400)

Biopsy of the mass with histopathology analysis showed diffuse architecture with mixture of large cleaved (centroblastic/centrocytic) and large noncleaved (centroblastic monomorphous) cells. The cytoplasm was scanty. The tumor cells did stain for CD20, CD79a and bcl2 protein. The proliferating fraction, as detected by Ki67 was high (more than 80% of neoplastic cells). The CD3 T-cell marker was negative. The immunohistochemical tests confirmed the diagnosis of large B-cell lymphoma (Figure 2). Based upon her clinical and histological presentation and radiographic images, the diagnosis of stage IE primary lymphoma of the cranial vault, according to the Ann Arbor classification system, was established. The patient received 4 cycles of rituximab 375 mg/m2 iv day 1 (d1), cyclophosphamide 750 mg/m2 iv d1, doxorubicin 50 mg/m2 iv d1, vincristine 1.4 mg/m2 iv d1, and prednisone 100 mg d1-5 combination chemotherapy (RCHOP) every 3 weeks, followed by involved field radiation. The patient had a remarkable improvement with a complete response after treatment and optimal local control after 9 months of follow-up.

## Discussion

The diagnosis of PBL is more definite in patients who present with isolated bone localisations of lymphoma without other sites of involvement. Most series show a male preponderance, higher likelihood of early-stage disease presentation, and a median age of presentation in the fifth or sixth decade. Involvement of the cranial vault is an unusual manifestation of non-Hodgkin’s lymphoma in patients without HIV infection. Only fourteen patients with non-HIV related primary cranial vault lymphoma have been reported in the literature [2-15]. Our patient was HIV negative and presented young age in comparison to the literature. Symptoms of lymphoma in the skull include a painless scalp lump, headache and focal neurological deficits [2,9,11,12]. In our case painless subcutaneous scalp lump was not associated with any neurological deficits. MRI of lymphomas cannot be specific because its appearance can mimic those of metastatic carcinoma, osteomyelitis, or meningioma, although the signal intensity of a lymphoma on MRI is non-specific, most reported cases of skull lymphomas were isointense on unenhanced MRI and showed marked enhancement after administration of contrast medium [13]. Because of the characteristic permeating growth pattern of lymphoma with large soft tissue component, bone destruction may not be seen as in present case. Besides, to confirm a diagnosis of cranial vault lymphoma, an IHC analysis is necessary. Most lymphomas of the cranial vault are B-cell non Hodgkin lymphoma [2-15], as in our case. In the literature review of 14 patients, complete remission was achieved in 75 % patients with median remission duration of 7 months (range, 1-72). Three of these patients were still disease free after 2 years. 60% received whole brain radiation alone, 40% received systemic chemotherapy plus whole brain radiation, and one received whole brain radiation and intrathecal methotrexate. However, combined modality treatment including anthracycline-based systemic chemotherapy and involved field radiation therapy is considered the standard for localized aggressive non-Hodgkin’s lymphoma [1]. Our case showed a good clinical outcome with complete response after a combination of sequential chemotherapy and radiotherapy.

Despite the increasing number of primary bone lymphoma and recent therapeutic advances, several questions still remain unanswered about the optimal management of these tumors. We believe that four cycles of a chemotherapeutic regimen (RCHOP) followed by involved field radiotherapy is the appropriate regimen for patients with primary localized cranial vault lymphoma. Furthermore, exclusive irradiation should be proposed for isolated cases. The prognosis is uncertain, but either direct cerebral invasion or leptomeningeal seeding should indicate a less favorable prognosis [13].

## Conclusion

This case demonstrates that PBL must be kept in mind in the differential diagnosis of primary lesions in the skull. Chemotherapy followed by involved field irradiation, appears to be an adapted therapy.

## Competing interests

The authors declare that they have no competing interests.

## Authors’ contribution

ZF: performed literature review, the composition of this case report and manuscript writing. IL-MA: conception and design collection and assembly of data. BK: analyse and interpretation of anatomo-pathology findings. SB-HM- HE: analyse and interpretation of data, manuscript writing. All authors read and approved the final version of the manuscript.
